# Development and Validation of a Tool to Improve Community Pharmacists’ Surveillance Role in the Safe Dispensing of Herbal Supplements

**DOI:** 10.3389/fphar.2022.916223

**Published:** 2022-07-04

**Authors:** Ammar Abdulrahman Jairoun, Sabaa Saleh Al Hemyari, Naseem Mohammed Abdulla, Moyad Shahwan, Maimona Jairoun, Brian Godman, Faris El-Dahiyat, Amanj Kurdi

**Affiliations:** ^1^ Health and Safety Department, Dubai Municipality, Dubai, United Arab Emirates; ^2^ School of Pharmaceutical Sciences, Universiti Sains Malaysia, Penang, Malaysia; ^3^ Pharmacy Department, Emirates Health Services, Dubai, United Arab Emirates; ^4^ School of Health and Environmental Studies, Hamdan Bin Mohammed Smart University (HBMSU), Dubai, United Arab Emirates; ^5^ Department of Environmental Health Sciences, Canadian University Dubai, Dubai, United Arab Emirates; ^6^ Department of Clinical Sciences, College of Pharmacy and Health Sciences, Ajman University, Ajman, United Arab Emirates; ^7^ Centre of Medical and Bio-allied Health Sciences Research, Ajman University, Ajman, United Arab Emirates; ^8^ Strathclyde Institute of Pharmacy and Biomedical Sciences (SIPBS), University of Strathclyde, Glasgow, United Kingdom; ^9^ Division of Public Health Pharmacy and Management, School of Pharmacy, Sefako -Makgatho Health Sciences University, Pretoria, South Africa; ^10^ Clinical Pharmacy Program, College of Pharmacy, Al Ain University, Al Ain, United Arab Emirates; ^11^ AAU Health and Biomedical Research Center, Al Ain University, Abu Dhabi, United Arab Emirates; ^12^ Center of Research and strategic studies, Lebanese French University, Erbil, Iraq; ^13^ Department of Pharmacology and Toxicology, College of Pharmacy, Hawler Medical University, Erbil, Iraq

**Keywords:** herbal supplement adulteration, community pharmacies, label accuracy, falsiability, dispensing, United Arab Emirates

## Abstract

**Background:** There has been an appreciable increase in the use of herbal supplements, including immune boosters, during the current COVID-19 pandemic. However, there are concerns with falsified herbal supplements.

**Objectives:** Developed a new questionnaire that can potentially help community pharmacists identify the extent of falsified herbal supplements.

**Methods:** A 9-month cross sectional study was conducted among 500 community pharmacies across United Arab Emirates. A new 5-factor, 24-itmes scale was developed based on current labelling requirements across countries and piloted. This included seven items on identified uses and contraindication, seven items on hazard identification, four items on product identity, three items on packaging and product insert and three items on product handling and storage. The face and content validity of the scale was assessed via the content validity index (CVI). Its construct validity was tested using an exploratory factor analysis (EFA) via principally component analysis (PCA). The model was subsequently confirmed through partial confirmatory factor analysis (PCFA). Its reliability was assessed via test-retest reliability, internal consistency, item internal consistency (IIC), and intraclass correlation coefficients (ICCs).

**Results:** The CVI of the finalized questionnaire was 0.843. The Kaiser-Meyer-Olkin measure of sampling adequacy was 0.891, and Bartlett’s test of sphericity indicated significance (*p*-value < 0.001). Confirmation of the subsequent 5-domains was achieved through PCFA using maximum likelihood analysis with oblimin rotation. The PCFA obtained values was 0.962 for NFI, 0.977 for CFI, and 0.987 for the Tucker Lewis Index. All values were greater than 0.95, and the RMSEA value was 0.03 (i.e., less than 0.06). Consequently, the model had a good fit. All domains demonstrated Cronbach’s alpha coefficients above 0.70, with 0.940 for the full instrument. Meanwhile, all items met the IIC correlation standard of ≥0.40. The instrument presented good ICC statistics of 0.940 (0.928–0.950) as well as statistical significance (*p* < 0.001). Community pharmacists with more than 10 experience years were more likely to identify falsified herbal supplements compared to those with 1–10 years experience (*p* < 0.001).

**Conclusion:** This study developed and validated a new instrument to identify safe herbal supplements, which should enhance the role of the community pharmacists in the safe and effective treatment of suitable patients with herbal supplements.

## Introduction

There is a rise in the use of herbal medicines across countries, which can also be referred to with terms including alternative medicines, botanical products, complimentary medicines, natural products and traditional medicines, with an appreciable proportion of the world’s population now using herbal medicines to treat some of their diseases (Byard et al., 2017; Alsayari et al., 2018; Eddouks et al., 2020; Geck et al., 2020; Market Data Forecast, 2021). Overall, it is expected that the herbal medicine market will grow at an annual compounded growth rate of over 7.2% between 2021 and 2026 due to their lower costs, accessibility, and belief that herbal medicines can promote healthier life-styles, treat diseases such as the metabolic syndrome and gastrointestinal problems, and typically have less side-effects than prescribed medicines, which can be a concern with the toxicity of some prescribed medicines (Calitz et al., 2015; Nuryunarsih, 2016; Alsayari et al., 2018; Bhat et al., 2019; Ekar and Kreft, 2019; Eddouks et al., 2020; Holleran et al., 2020; Market Data Forecast, 2021). This is despite concerns with the evidence base of some herbal medicines (Geck et al., 2020; Holleran et al., 2020; Popattia et al., 2021), as well as potentially adverse effects including increased liver and kidney toxicity exacerbated by some herbal medicines containing heavy metals and naturally occurring organic toxins (Calitz et al., 2015; Brown, 2017; Byard et al., 2017; Kum et al., 2021). However, there are relatively infrequent reports of adverse reactions with herbal medicines across countries (Di Lorenzo et al., 2015). In addition, the quality of some herbal medicines can also vary substantially impacting on their potential toxicity as seen recently with Danshen (Kum et al., 2021). These concerns though have not impacted on the growth of this market.

However of concern are the differences in the legislation surrounding the regulations and sale of herbal medicines across countries (Dwyer et al., 2018; Bhat et al., 2019). In the United States, for instance, the Food and Drug Administration (FDA) requires that herbal supplements labels should include information pertaining to its name, name and address of the manufacturer, and a complete list of the ingredients including the amount of each active substance (@mayoClinic, 2021). This though is not universal, and it is increasingly likely that these issues will need to be addressed to enhance the safe use of herbal medicines as their evidence base grows. This may well require changes in culture as well as harmonized standards on key issues such as quality control as well as evidence standards across countries as their use grows (Geck et al., 2020; Xiong et al., 2021). The same issues are seen with the use of dietary supplements where again there can be both benefits and concerns (Khan et al., 2019; Jairoun et al., 2020a; Parks et al., 2020).

Another key concern is that the increased use of herbal medicines has been accompanied by their adulteration, which includes increased use of synthetic compounds to increase profit levels; however, this leads to concerns with quality control unless proactively addressed (Ekar and Kreft, 2019; Enioutina et al., 2020; Kum et al., 2021; Okaiyeto and Oguntibeju, 2021). Alongside this, there have also been an increase in the extent of falsified medicines, including herbal medicines, for both life style and health-related conditions across countries (BottomLineInc, 2014; Newsmax_Fake_2015; Nuryunarsih, 2016; Ekar and Kreft, 2019). Examples of include adulteration or contamination of herbal weight-loss products as well as herbal preparations for inflammatory diseases, blood pressure control and the treatment of certain central nervous system disorders including depression (Newmaster et al., 2013; Ozdemir et al., 2013; Little, 2014; Wiest et al., 2014; Frommenwiler et al., 2016; Khan et al., 2016; Raclariu et al., 2017; Ekar and Kreft, 2019).

Community pharmacists play a key role in patient care across countries, enhanced by the fact that they are often being the first point of healthcare professional contact for patients for most non-severe conditions, which includes patients with suspected COVID-19 where hospitalization is not required (Marković-Peković et al., 2017; Cadogan and Hughes, 2021; Hedima et al., 2021; Kibuule et al., 2021). Encouragingly, they also typically appear to have good knowledge regarding the indications, side-effects and contraindications of herbal medicines (Alkharfy, 2010; Asmelashe Gelayee et al., 2017; Alsayari et al., 2018). However, this is not always the case as seen in a recent study conducted in Palestine (Shraim et al., 2017) and in the US where practicing pharmacists identified a number of barriers to enable safe and appropriate dispensing of nutritional and herbal supplements (Ung et al., 2019). Identified barriers included a lack of education and training surrounding nutritional and herbal supplements as well as concerns regarding regulatory standards and the efficacy and safety of these supplements (Ung et al., 2019) Harnett et al. (2019) also identified key concerns among pharmacists to enhance the use of herbal medicines. This included their education and training to ensure high standards regarding the safety and quality assurance of supplements (Harnett et al., 2019). However, until recently no formal framework appears to be in operation to describe the responsibilities of community pharmacists in dispensing herbal medicines, although this is now changing (Popattia et al., 2018; Popattia et al., 2021; Popattia and La Caze, 2021).

In view of these concerns, there is a need to develop a new approach that can help community pharmacists identify the quality of the herbal supplements they dispense. This includes identifying falsified herbal supplements based on the regulatory label requirements of the European Union, the United States FDA and the Dubai Municipality Health and Safety Department, United Arab Emirates (UAE) (FDA, 2005; European Commission, 2014; The Guardian, 2015; Government of Dubai, 2022). This builds on initiatives across countries, including African countries, to reduce the extent of falsified medicines (WHO, 2020; Ogunleye et al., 2020) as well as initiatives such as DNA barcodes or amplicon metabarcoding (AMB) to detect contaminated products alongside routine surveillance activities (Wallace et al., 2012; Newmaster et al., 2013; Little, 2014; Raclariu et al., 2017).

Consequently, to address this need whilst techniques such as AMB develop, we compiled and evaluated different label requirements of dietary herbal supplement products across different Health Regulatory Authorities, including US, EU and the UAE. The objective is to develop and validate a potential novel self-reporting tool to improve the routine identification of falsified dietary herbal supplements among community pharmacist in the UAE and wider. Such tools can potentially be useful, along with addressing pertinent education and training requirements, to promote the safe and appropriate dispensing of herbal supplements in community pharmacies (Harnett et al., 2019; Ung et al., 2019).The findings can subsequently be used across countries to tackle this increasingly important issue.

## Methods

### Study Design and Setting

This study was a descriptive analytical cross-sectional study conducted over 9 months from January 2021 to September 2021 among community pharmacies across the UAE, which included Abu Dubai, Dubai and the Northern Emirates.

This included a pilot study to test the questionnaire before full roll out. Subsequently, evaluate the face and content validity of the questionnaire using standard approaches for use in the future. We adopted a similar methodology to our recently published paper regarding falsified hand sanitisers at the start of the pandemic (Jairoun et al., 2020b).

English was chosen as the language for the questionnaire as it is the international scientific language and the common language for scientific research scientists globally. In addition, potentially enhancing the usefulness of any tool subsequently developed.

**FIGURE 1:**
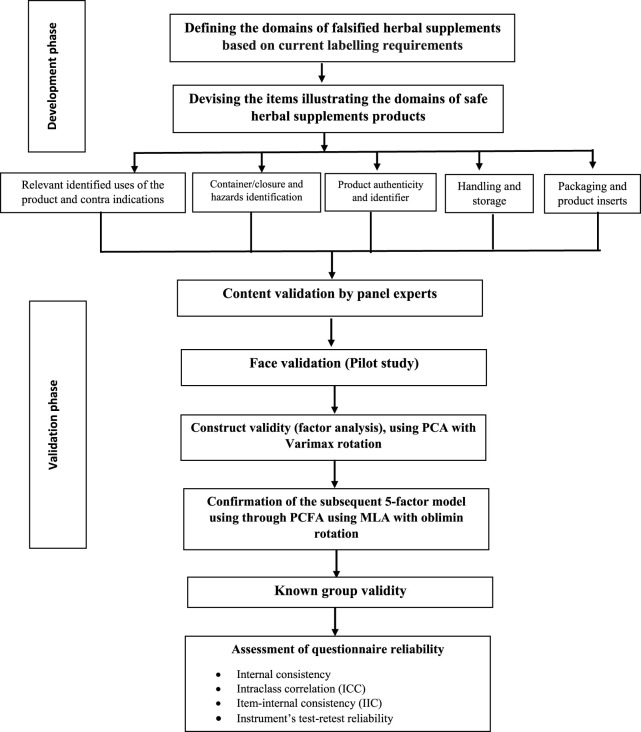
Flow chart for development and validation process.

Figure 1 describes the development and validation process.

### Study Participants (Inclusion and Exclusion Criteria)

The study subjects were chosen based on pre-specified inclusion and exclusion criteria. The inclusion criteria were community pharmacists who had 3 months’ professional experience or more and were registered with one of the three regulatory bodies, i.e. the Ministry of Health, the Health Authority Abu Dhabi (HAAD), or Dubai Health Authority.

### Pilot Testing

A pilot study was undertaken to test the face validity of the developed tool among 22 community pharmacists, whose data were subsequently excluded from the final analysis. The pilot study was undertaken between 12 January 2021 and 22 January 2021. The outcomes of the pilot study were employed to calculate the sample size needed for the main research and to check its reliability.

The questionnaire was sent to 25 purposively selected community pharmacists from which there were 22 respondents, yielding a response rate of 88%.

### Research Instrument Development and Conceptualization

#### Face and Content Validity

The questionnaire was developed based on current labelling requirements of the European Union and the United States FDA, and the Dubai Municipality Health and Safety department (Figure 1). In their labeling requirements, key information including the Brand name, the name of the manufacturer, the country of origin, the ingredients, product names, pack sizes, Production & Expiration Dates, Storage Conditions, Dosage and Instruction of Use, Product indications/intended use, any Health warnings, Identification Code (Barcode) and Batch Number. All required to be printed on dietary herbal supplement product labels. The labelled indications are designed to provide users with a clear identification of the functioning and proper use of any dietary herbal remedy, as well as to safeguard the user from commercial considerations and, most importantly, from safety concerns. Labelling should also provide the information required for easy tracing of product data and all toxicologically relevant data. Under normal presentation conditions, the lettering used for dietary herbal supplement product labelling must be permanent, easy to read, and be readily apparent, e.g., in terms of contrast with the background and size.

The initial draft of the questionnaire was subjected to face and content validity by a panel of experts, which comprised two community pharmacists, two regulatory pharmacists, three academics and two industrial pharmacists.

The content validity index (CVI) and ratio (CVR) were calculated by asking every expert to mark each item in the questionnaire as essential or non-essential. A CVR of 0.78 or higher could be considered as evidence of good content validity. If an item does not reach this threshold, it would normally be deleted from the final instrument. Following this, the CVI was obtained by calculating the mean of the CVR values for all items meeting CVR threshold of 0.78 and retained for the final instrument (Lawshe, 1975; Rungtusanatham, 1998).

#### Construct Validity

Construct validity was tested using an exploratory factor analysis (EFA). The factor analysis was conducted by a principal component analysis (PCA), followed by Varimax rotation with Kaiser-Meyer-Olkin (KMO) and Bartlett’s test of sphericity to determine the number of factors. Eigenvalues of one and Items loaded of at least 0.40, with no cross-loading of items above 0.40 were used to satisfy the criteria of construct validity (Straub and Gefen, 2004).

The model was subsequently confirmed through partial confirmatory factor analysis (PCFA) using maximum likelihood analysis (MLA) with oblimin rotation. The incremental fit indices, namely the comparative fit index (CFI), normed fit index (NFI) and the Tucker Lewis index (TLI), were subsequently calculated. The absolute fit index, i.e., root mean square error of approximation (RMSEA), was also calculated and reported (Cohen, 1988; Devellis, 1991).

#### Internal Consistency and Reliability Analyses

Internal consistency and test–retest reliability were assessed using Cronbach’s alpha and intraclass correlation coefficients (ICCs), respectively. The criteria of accepting Cronbach’s alpha are equal to or above 0.70 (Nunnally, 1978). The criteria for interpretation of ICCs are based on Rosner, i.e. ICC <0.40 is seen as poor agreement, 0.40 ≤ ICC <0.75 is seen as fair to good agreement, and an ICC ≥0.75 represents excellent agreement (Rosner, 2000).

The Item internal consistency (IIC) was measured by the Pearson correlation, which assess the relationship of each item to its hypothesized factor or domain. The IIC rule requires that the item should correlate r ≥ 0.4 with its adjusted scale score (Ware and Gandek, 1998). The test-retest reliability between two time-points was assessed after a gap of 6 weeks through Pearson’s correlation coefficient (ρ). A value of (ρ) more than 0.75 and *p*-value < 0.05 was considered as a significantly strong correlation (Cohen, 1988; Devellis, 1991).

#### Known Group Validation

We hypothesized that participants with greater experience would be more able to identify falsified herbal supplement compared with their counterparts. Consequently for this study, participants were categorized into two groups, namely those with 1–10 years of experience and those with >10 years of experience. The known group validity was evaluated through one-way ANOVA test and a *p*-value less than 0.05 was considered acceptable.

### Sample Size Calculation

The sample size calculation for the full study was based on the answers to the question “Do you know how to identify herbal supplements safety based on the product label?” in the pilot study.

According to the pilot study, the proportion of people who answered yes to this question was 44%. The alpha level was set at 5%, giving a 95% confidence interval. Precision (D) for the 95% confidence interval was fixed at 5% so that the 95% CI would have a maximum width of 10%. On the basis of these assumptions, a sample size n of 541 was required, assuming that nonresponse rates would be approximately 30%.

### Sampling Technique

To ensure representativeness, this study used a simple random sampling technique. In 2010, it was estimated that a total of 2000 community pharmacies are practicing across the UAE (Jairoun et al., 2020b). The contact details and locations of community pharmacies in the areas chosen for study were taken from local business directories and the Yellow Pages.

After the sampling, the randomly selected pharmacies were stratified into groups or strata based on the community pharmacies’ locations. Accordingly, three strata were identified, as follows: community pharmacies located in Abu Dubai, community pharmacies located in Dubai, and community pharmacies located in the Northern Emirates.

Once pharmacies had been selected up to a total of 541, Excel software was used to record all related data to serve as a sampling frame, reporting the name, type, location, email address, and phone number of each pharmacy. Each pharmacy was given an ID number, after which all the listed pharmacies were subjected to a simple random sample selection process. Community pharmacies selected for inclusion were categorized by type and location and subsequently visited.

### Data Collection

Selected community pharmacies across, Abu Dubai, Dubai and the Northern Emirates were visited between 27 January 2021 and 27 September 2021. The researchers explained the purpose of the research to the pharmacists and noted their email addresses. Face-to-face interviews were subsequently undertaken using the structured questionnaire, based on the pilot study, among those who agreed to participate.

### Statistical Analysis

Data analysis was performed using SPSS version 24. The participants answered the questionnaire where pertinent using a 5-point Likert scale (0 = “Never”, 1 = “Rarely”, 2 = “Sometimes”, 3 = “Often” and 4 = “Always”) (Supplementary file). Descriptive statistics, including frequencies and percentages for categorical variables and mean (SD) for continuous variables, were used to summarize the demographic and baseline characteristics of the study sample. To test the known group validity, the association between the falsified herbal supplements identification score (outcome variable) and year of experience (independent variables) was tested using one-way ANOVA. Statistical significance was considered at *p*-value less than 0.05.

### Ethical Consideration

The study was approved by the Institutional Ethical Review Committee of Ajman University P-H-S-2021-2-11. Before data collection, the purpose of the survey was explained to all potential community pharmacists. Community pharmacists were also informed that completion and submission of the questionnaire would be undertaken upon their consent. All participants signed the informed consent. No participant identities were recorded and confidentiality was guaranteed.

## Results

### Demographic Details of the Participants

A total of 500 community pharmacists participated in the full study out of a possible total of 541 pharmacists and completed the questionnaire. Of the total participants, 328 (65.6%) were female and 172 (34.4%) were male. The average age of the participant community pharmacists was 32.2 ± 6.8 years.

Most of the participant community pharmacists were Arabic (Table 1) with a minority from the Emirati (14.0%), Asia (6.0%) and Africa (4.4%). Among the total number of community pharmacists taking part, 188 (37.6%) had between 1 and 10 years’ experience and 312 (62.4%) had more than 10 years’ experience (Table 1).

**TABLE 1:** Demographic information (*n* = 500).

Demographics	Groups	Frequency	Percentage
Age (mean ± S.D)	32.2 ± 6.8		
Gender	Female	328	65.6
	Male	172	34.4
Age groups	18–24 years	396	79.2
	25–34 years	52	10.4
	≥35 years	52	10.4
Nationality	African	22	4.4
	Arabic	364	72.8
	Asian	30	6.0
	Emirati	70	14.0
	Western	14	2.8
Years of Experience	1–10 years	188	37.6
	More than 10 years	312	62.4

### Validation Analysis

#### Face/Content Validity

The first draft of the instrument comprised 31 items. After the draft was evaluated by the expert panel, ten of these items were modified based on their feedback. We subsequently determined that seven items did not meet the minimum CVR of 0.78, and these were subsequently eliminated from the scale. The final 24-item scale had a CVI of 0.843.

#### Construct Validity (Factor Analysis)

As mentioned, EFA using PCA with varimax rotation was used to assess the structure of the instrument’s factors. The KMO measure of sampling adequacy was 0.891, and Bartlett’s test of sphericity indicated significance (*p*-value< 0.001).

**FIGURE 2:**
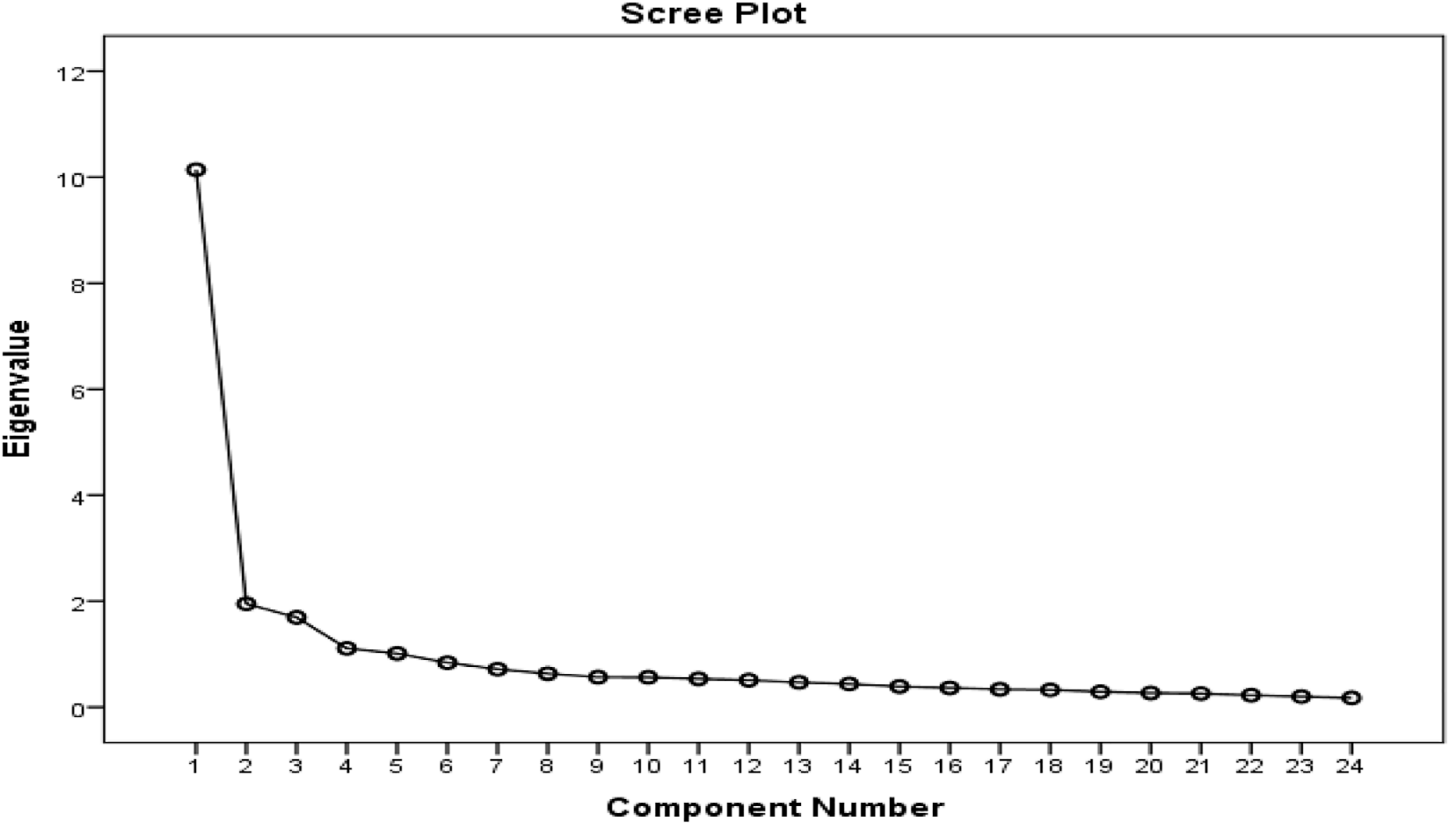
Scree plot and component factors resulting from the PCA.

A five-factor solution was determined to have eigenvalues above one and accounted for 66.3% of the variance with factor 1 presenting 42.3% and factors 2, 3, four and five constituting 8.1, 7.1, 4.6 and 4.2% respectively. Items that factor loaded >0.4 onto one component and demonstrated non-salient loading <0.4 on another component were considered to be a single factor. A clear factor structure was thereby obtained (Table 2; Figure 2).

**TABLE 2:** Content validity ratio and factor structure emerging from principle factor analysis.

	Item content	CVR	Component					Communalities
			1	2	3	4	5	
Factor 1	Restrictions on use by specific groups of consumers e.g. during pregnancy, during breastfeeding, for children under 3 years	0.83	0.776	0.208	0.128	0.019	0.264	0.731
Relevant identified uses of the substance or mixture and uses advised against	Relevant identified used, recommended use and restrictions on use	0.87	0.751	0.203	0.027	0.218	0.171	0.683
	Warning and cautions are clearly indicated on the product label	0.83	0.728	0.216	0.101	0.125	0.254	0.667
	Information on hazardous ingredients e.g. contains X - where X is a component that requires warning	0.81	0.675	0.229	0.336	0.226	0.035	0.673
	Information on the package insert match with the information on the product container	0.77	0.635	0.065	0.309	0.337	0.082	0.623
	Warning of Keep out of reach of children	0.74	0.618	0.099	0.198	0.180	0.382	0.643
	List of ingredients or reference to enclosed or attached information about list of ingredients	0.92	0.517	0.149	0.368	0.270	0.225	0.602
Factor 2	Dietary supplements container is safely sealed	0.79	0.161	0.782	0.008	0.093	0.339	0.762
Container/Closure and Hazards identification	Dietary supplements container and closure protect the product from the outside environment	0.83	0.099	0.731	0.116	-0.075	0.345	0.683
	The premises and shops selling the dietary supplements assure that the dietary supplements meet the proper specifications throughout their shelf life	0.81	0.327	0.686	0.259	0.062	0.107	0.659
	The container and the closure are appropriate for the dietary supplement product inside	0.86	0.196	0.684	0.300	0.125	0.139	0.631
	Information on the outer box of the dietary supplements match with the label on the container	0.74	0.125	0.645	0.310	0.295	-0.046	0.618
	All information on the dietary supplement labels are legible and indelible	0.93	0.353	0.486	0.375	0.254	-0.144	0.587
	The product labeled as Dietary supplements or food supplements	0.84	0.059	0.431	0.396	0.428	0.347	0.650
Factor 3	Trade name is spelt correctly	0.81	0.131	0.264	0.779	0.151	0.156	0.740
Product authentic and identifier	The manufacturer’s name and logo are legible and correct	0.88	0.141	0.139	0.676	0.337	0.158	0.635
	The active ingredient name is spelt correctly (scientific name/generic name)	0.92	0.335	0.235	0.664	0.058	0.052	0.614
	The dietary supplement product is registered in the country by the concerned Regulatory Authority	0.87	0.170	0.395	0.563	0.117	0.134	0.611
Factor 4	The size and Volume are clearly indicated in the product label	0.82	0.085	0.001	0.283	0.732	0.211	0.667
Packaging and product insert	The number of dosage units listed on the label matches with the number of dosage units stated on the container	0.78	0.346	0.107	0.063	0.716	0.003	0.719
	The dosage stated on the label is appropriate for the dietary supplement product in this form and strength	0.86	0.317	0.248	0.154	0.674	0.070	0.645
Factor 5	The manufacturing and expiry dates are clearly indicated on the label	0.84	0.237	0.315	-0.108	0.149	0.728	0.719
Handling and storage measure	The storage conditions are indicated on the label	0.93	0.341	0.140	0.316	0.049	0.685	0.707
	The product has been properly stored	0.96	0.265	0.188	0.294	0.129	0.655	0.638

Confirmation of the subsequent 5-factor model was achieved through PCFA using MLA with oblimin rotation. The KMO was 0.882, and Bartlett’s test of sphericity demonstrated significance (*p*-value<0.001). The distribution curve of the non-salient factor loading was normal with a mean value of 0.1. The null model χ2 was 8239.25, and the χ2 of the implied model was 945.57. The PCFA obtained values of 0.962 for NFI, 0.977 for CFI, and 0.987 for TLI; all values were greater than 0.95, and the RMSEA value was 0.03 (i.e., less than 0.06). Consequently, the model had a good fit.

### Reliability Analysis

Internal consistency (Cronbach’s alpha), IIC, and ICC were used to assess the reliability of the developed instrument. All factors demonstrated Cronbach’s alpha coefficients above 0.70, with 0.940 for the full instrument. Meanwhile, all items met the IIC correlation standard of ≥0.40. Finally, the instrument presented good ICC statistics of 0.940 (0.928–0.950) as well as statistical significance (*p* < 0.001).

The ICC for the factors ranged between 0.745 and 0.897. The reliability of factor 1 was reported at 0.897 with 95% confidence interval of (0.876–0.915). Factor 2 had an alpha value of 0.872, ICC = 0.846–0.895 for 95% CI. Factor 3 had an alpha value of 0.817, ICC = 0.778–0.851 for 95% CI. Factor 4 had an alpha value of 0.745, ICC = 0.778–0.844 for 95% CI. Factor 5 had an alpha value of 0.782, ICC = 0.731–0.825 for 95% CI. Further details are given in Table 3.

**TABLE 3:** Reliability assessment criteria of the study scale (*n* = 500).

Subscale	No. of items	Mean ± SD	Cronbach’s α	IIC	ICC (95%CI)
Factor 1	7	47.23 ± 6.8	0.897	0.765–0.797	0.897 (0.876–0.915)
Factor 2	7	21.8 ± 6.6	0.872	0.684–0.803	0.872 (0.846–0.895)
Factor 3	4	11.5 ± 4.1	0.817	0.782–0.854	0.817 (0.778–0.851)
Factor 4	3	9.3 ± 3.1	0.745	0.778–0.844	0.745 (0.685–0.796)
Factor 5	3	10.5 ± 2.9	0.782	0.810–0.848	0.782 (0.731–0.825)
Total	24	76.5 ± 19.4	0.940	-----------------	0.940 (0.928–0.950)

Abbreviations: SD, (standard deviation); IIC, item-internal consistency; ICC, intraclass correlation (consistency ICC from 2-way mixed model).

The instrument’s test-retest reliability was measured through a correlation between the participants’ identification scores for counterfeit and substandard herbal supplements at time points 1 and 2, with a 3-week gap; the correlation coefficient was 0.872 (*p*-value < 0.01).

### Known Group Validity

As shown in Table 4, the known group validity was assessed by one way ANOVA. A statistically significant relationship was seen between the experience in years and the reported falsified herbal supplement identification scores.

**TABLE 4:** Substandard and falsified herbal supplements identification scores by experience years.

Experience years	Herbal supplements safety identification scores			
	Mean	± SD	95% confidence interval	
			Lower limit	Upper limit
1–10 years	79.8	23.6	75.08	86.66
More than 10 years	83.7	22.1	80.12	87.41

Those participants who had more than 10 years of experience more likely to identify falsified herbal supplements compared to those who had only 1–10 years of experience (*p* < 0.001).

## Discussion

We believe this is one of the first published studies to develop a method (an instrument) that can help community pharmacists identify falsified and substandard herbal supplements within their pharmacies Supplementary Appendix 1. This is important given the anticipated growth in sales of herbal supplements driven by a likely increase in the number of patients seeking herbal supplements from community pharmacies in the future, exacerbated by the current COVID-19 pandemic with patients seeking herbal medicines in the absence of proven effective pharmaceuticals assisted by studies demonstrating their benefits (Alsayari et al., 2018; Mirzaie et al., 2020; Silveira et al., 2020; Brendler et al., 2021; Feng et al., 2021; Khanna et al., 2021; Market Data Forecast, 2021; Demeke et al., 2021; Sefah et al., 2021a, 2021b). In addition, growing reports of falsified or adulterated herbal supplements, with concerns for the health of patients (BottomLineInc, 2014; Newsmax Fake, 2015; Raclariu et al., 2017; Kum et al., 2021).

Encouragingly, the content validity index yielded a satisfactory value of 0.843, with the Cronbach’s alpha seen as good (0.940), with the alpha values for the five components of the scale also seen as very acceptable (0.745–0.897). The instrument’s temporal stability was also seen as good as evidenced by the results of test-retest examinations.

Five different components of the conceptual model were identified as having eigenvalues larger than one, as predicted by the model, which is also encouraging. This accounted for 66.3 percent of the variation. The correlation matrix was factorable, as evidenced by the KMO value of 0.891 and the statistical significance of Bartlett’s test of sphericity (*p* < 0.001). The PCFA analysis verified the 5-factor model solution and showed a strong match. Consequently, this measurement scale was seen as robust in identifying key areas. These include the manufacturer’s name and logo, the names of active ingredients, if the herbal supplement product was registered with the relevant Regulatory Authority as well as double-checking critical package and product insert information. Encouragingly as well, the instrument could distinguish across groups with varying experience levels, which is to be expected.

The length of the instrument and the time it took to complete the questionnaire were viewed as positive points, which resulted in a high response rate and excellent levels of respondent accuracy.

Consequently, we believe this tool can help advance the role of community pharmacists in the appropriate and safe dispensing of herbal supplements, building on the comments of Harnett et al. (2019). Future studies can research the use of the tool in community pharmacies to assess whether falsified or adulterated herbal supplements can readily be detected whilst relying on surveillance using for instance DNA barcodes or AMB techniques to detect contaminated or falsified products (Wallace et al., 2012; Newmaster et al., 2013; Little, 2014; Raclariu et al., 2017).

Such studies can also help to develop a more complete map of the routes via which fake herbal supplements are trafficked including concerns with free trade zones and their implications (OECD and Office EUIP, 2018), as well as a better understanding of their effects on consumers, the industry, and key state-level groups. Furthermore, our tool should help community pharmacists address one of their key ethical responsibilities, that is being vigilant of any harm associated with herbal supplements and subsequently intervening in the case of any perceived significant risk of harm related to the dispensing of herbal supplements (Popattia et al., 2021; Popattia and La Caze, 2021). Alongside this, the mass sale of counterfeit products via internet shopping channels also needs to be addressed However, this is outside the scope of the current project.

Overall, we believe we have developed a robust and valid tool building on the Lome and other initiatives for falsified medicines (WHO, 2020b). This is because of the many stages employed in its development. Consequently, we are confident in using the questionnaire in future studies to assess the extent of fraudulent herbal supplements in UAE and across countries in the future. The findings should enhance the role of the community pharmacists in treating suitable patients with herbal supplements, and being confident in the product they dispense recognising their legal requirements. This will be important for future patient safety and sales. We will also now work with Governments and Pharmacy Groups in UAE and wider to see how we can assist with identifying falsified herbal supplements in the future based on our study findings.

We are aware of a number of limitations with our study. Firstly, we conducted this study in only one country. Secondly, we have not tested the tool we have developed in practice. However, we believe that in view of our robust methodology we would expect the tool to work in practice and across countries since these problems are not confined to community pharmacists in UAE.

## Conclusion

In conclusion, this study developed and validated a new scale for safe dispensing of the herbal supplement formulations. The final instrument appeared concise and easy to administer, making it appropriate for use in community pharmacies. Ultimately, this tool can facilitate collaboration between community pharmacist, health regulatory authorities and inspection authorities to identify falsified supplements. As a result, this study could provide decision-makers in the public and private sectors with informed insights into the worldwide counterfeit herbal supplement trade. This will enable them to create policy solutions that are both relevant and effective in the future.

## Data Availability

The original contributions presented in the study are included in the article/Supplementary Material, further inquiries can be directed to the corresponding authors.

## Abbreviations

AU, Ajman University; CFI, comparative fit index; CVI, content validity Index; CVR, content validity ratio; EFA, Exploratory factor analysis; ICCs, intraclass correlation coefficients; IIC, item internal consistency; KMO, Kaiser-Mayer-Olkin; NFI, normed fit index; PCA, principal component analysis; PCFA, partial confirmatory factor analysis; RMSEA, root mean square error of approximation; TLI, Tucker-Lewis index; UAE, United Arab Emirates.
